# Grape pomace extract exerts antioxidant effects through an increase in GCS levels and GST activity in muscle and endothelial cells

**DOI:** 10.3892/ijmm.2015.2246

**Published:** 2015-06-15

**Authors:** NIKOLAOS GOUTZOURELAS, DIMITRIOS STAGOS, ANASTASIA HOUSMEKERIDOU, CHRISTINA KARAPOULIOU, EFTHALIA KERASIOTI, NEKTARIOS ALIGIANNIS, ALEXIOS L SKALTSOUNIS, DEMETRIOS A SPANDIDOS, ARISTIDIS M TSATSAKIS, DEMETRIOS KOURETAS

**Affiliations:** 1 Department of Biochemistry and Biotechnology, University of Thessaly, Larissa 41221, Greece; 2 Division of Pharmacognosy and Natural Products Chemistry, School of Pharmacy, University of Athens, Athens 15771, Greece; 3 Laboratory of Clinical Virology, University of Crete, Medical School, Heraklion 71409, Greece; 4 Department of Forensic Sciences and Toxicology, Medical School, University of Crete, Heraklion 71003, Greece

**Keywords:** grape pomace extract, oxidative stress, muscle cells, endothelial cells, glutathione, antioxidants

## Abstract

In a previous study, we demonstrated that a grape pomace extract (GPE) exerted antioxidant activity in endothelial (EA.hy926) and muscle (C2C12) cells through an increase in glutathione (GSH) levels. In the present study, in order to elucidate the mechanisms responsible for the antioxidant activity of GPE, its effects on the expression of critical antioxidant enzymes, such as catalase (CAT), superoxide dismutase (SOD)1, heme oxygenase 1 (HO-1) and gamma-glutamylcysteine synthetase (GCS) were assessed in EA.hy926 and C2C12 cells. Moreover, the effects of GPE on CAT, SOD and glutathione S-transferase (GST) enzymatic activity were evaluated. For this purpose, the C2C12 and EA.hy926 cells were treated with GPE at low and non-cytotoxic concentrations (2.5 and 10 *µ*g/ml for the C2C12 cells; 0.068 and 0.250 *µ*g/ml for the EA.hy926 cells) for 3, 6, 12, 18 and 24 h. Following incubation, enzymatic expression and activity were assessed. The results revealed that treatment with GPE significantly increased GCS levels and GST activity in both the C2C12 and EA.hy926 cells. However, GPE significantly decreased CAT levels and activity, but only in the muscle cells, while it had no effect on CAT levels and activity in the endothelial cells. Moreover, treatment with GPE had no effect on HO-1 and SOD expression and activity in both cell lines. Therefore, the present results provide further evidence of the crucial role of GSH systems in the antioxidant effects exerted by GPE. Thus, GPE may prove to be effective for use as a food supplement for the treatment of oxidative stress-induced pathological conditions of the cardiovascular and skeletal muscle systems, particularly those associated with low GSH levels.

## Introduction

Free radicals are produced in living organisms during normal metabolism (e.g., the reactions of mitochondrial respiratory chain and cytochrome P450), inflammation, phagocytosis and other physiological processes (1,2). The most important category of free radicals is constituted by reactive oxygen species (ROS), such as superoxide radical anion (O_2_^•−^), hydroxyl radical (OH^•^), peroxyl radical (RO^•^_2_) and hydrogen peroxide (H_2_O_2_) (2). An amount of ROS is necessary for a number of functions of an organism, including phagocytosis (3), intracellular signaling (2), cell proliferation, and apoptosis (4). However, the excessive production of ROS may lead to oxidative stress, a pathophysiological condition which has been implicated in the oxidative damage of macromolecules (lipids, protein and DNA) (2,5), immune dysfunction (6), muscle damage (7) and fatigue (8).

Oxidative stress occurs frequently in muscle tissue exposed to ROS production. For example, during intense exercise there is a high rate of O_2_ consumption in skeletal muscle that may cause incomplete O_2_ reduction and electron leakage from the electron transfer chain, as well as the extra-mitochondrial production of ROS, leading to the generation of ROS and oxidative stress. These effects in turn result in muscle fatigue and cell damage and apoptosis (9,10).

Moreover, oxidative stress-induced damage of the vascular endothelium may lead to the development of various diseases (11). A redox imbalance in endothelial cells results in the surface expression of different endothelial cell adhesion molecules, suggesting that oxidative stress induces acute and chronic phases of leukocyte adhesion to the endothelium (12,13). It has also been shown that the interaction between ROS and nitric oxide (NO) sets off a vicious circle, which results in further endothelial activation and inflammation (11). Furthermore, ROS, such as H_2_O_2_ can diffuse throughout endothelial cells and react with cysteine groups in proteins to modify their function (14). Thus, under conditions of oxidative stress, endothelial cells can lose integrity, progress to senescence and detach into the circulation (15).

However, every living organism has antioxidant mechanisms, including both enzymatic and non-enzymatic with which to counteract the overproduction of free radicals (2). Moreover, we have previously demonstrated that the supplementation of antioxidants through diet may be used to reduce the detrimental effects of oxidative stress on human health (16–18). Some of the most well known food sources of antioxidants are grapes and wine (19). Our research group has conducted several studies on the antioxidant properties of grapes which are attributed mainly to their polyphenolic content (20–24). In another previous study of ours, we demonstrated that a grape pomace extract (GPE) rich in polyphenols derived from pomace, a by-product of the wine-making process consisting of peels, seeds and stems, reduced oxidative stress in muscle and endothelial cells mainly through an increase in glutathione (GSH) levels (25). Thus, in the present study, the effects of GPE on enzymes which are crucial for GSH metabolism, such as gamma-glutamylcysteine synthetase (GCS) and glutathione S-transferase (GST) were investigated in endothelial and muscle cells. Moreover, the effects of GPE on other critical antioxidant enzymes, such as catalase (CAT), superoxide dismutase (SOD) and heme oxygenase 1 (HO-1) were examined in endothelial and muscle cells. The investigation of the effects of GPE on antioxidant enzymes at the cellular level may help to elucidate the molecular mechanisms through which it exerts its antioxidant effects. The understanding of these mechanisms provides valuable knowledge which may aid in the preparation of plant extracts aimed to be used as food supplements.

## Materials and methods

### Extract preparation

The grape extract examined was obtained from Batiki Tyrnavou variety (a red grape variety grown in Central Greece) of the *Vitis vinifera* species. The isolation of the extract was carried out as previously described (26). In brief, the raw material was dried in a shady, well-ventilated environment and extracted using ethanol (96%) at 50°C for 4 h. Following filtration, the solvent was evaporated under reduced pressure, and the residue (i.e., GPE) was kept at −20°C until further use.

The polyphenolic composition of the extract identified by a liquid chromatography/high resolution mass spectrometry (LC-HRMS) method in positive and negative mode has been reported previously (26). Thus, the extract was composed of flavan-3-ols (catechin and epicatechin), anthocyanidins, (cyanidin, malvidin, delphinidin and petunidin), anthocyanins (myrtillin, kuromanin, oenin and peonidin-3-O-glucoside) and flavonols (quercetin), phenolic acids (gallic acid and caftaric acid). Moreover, the total polyphenolic content (TPC) of the extract was evaluated and found equal to 648 mg of gallic acid per g of extract (26).

### Cell culture conditions

The C2C12 muscle cells were a gift from Professor Koutsilieris (National and Kapodistrian University of Athens, Athens, Greece) and the EA.hy926 cells were from Professor Koukoulis (University of Thessaly, Larissa, Greece). All the cells were cultured in normal Dulbecco’s modified Eagle’s medium (DMEM), containing 10% (v/v) fetal bovine serum (FBS), 2 mM L-glutamine, 100 U/ml of penicillin and 100 U/ml of streptomycin (all from Gibco, Paisley, UK) in plastic disposable tissue culture flasks at 37°C in 5% carbon dioxide.

### Treatment of the cells with GPE

The C2C12 and EA.hy926 cells were seeded in culture flasks and incubated for 24 h. The medium was then removed and replaced with serum-free medium containing GPE at non-cytotoxic concentrations (2.5 and 10 µg/ml for the C2C12 cells and 0.068 and 0.025 *µ*g/ml for the EA.Hy926 cells) followed by incubation for 3, 6, 12, 18 and 24 h. In a previous study of ours, it was shown that these concentrations were non-cytotoxic to the C2C12 and EA.hy926 cells (25). Untreated cells were used as controls.

Following treatment, the cells were lysed in radio-immuno-precipitation buffer [RIPA buffer; 50 mM Tris-HCl, 150 mM NaCl, 0.25% SDS, 0.25% sodium deoxycholate and 1 mM ethylenediaminetetraacetic acid (EDTA), pH 8.0] containing protease inhibitors (Complete™ mini protease inhibitors; Roche, Basel, Switzerland) for the preparation of the whole cell lysate. The cell lysates were then centrifuged at 16,250 × g for 20 min at 4°C. The supernatant was collected, and the amount of protein was then determined using Bradford reagent (Sigma-Aldrich Ltd., Munich, Germany). For the preparation of the cytosolic lysate, the cells were lysed in cytosolic lysis buffer [10 mM 4-(2-hydroxyethyl)-1-piperazineethane sulfonic acid (HEPES)-potassium hydroxide (KOH) pH 7.9, 1.5 mM MgCl_2_, 10 mM potassium chloride (KCl), 0.5 mM dithiothreitol (DTT) and 0.5% NP-40] to which protease inhibitors are added (Complete™ mini protease inhibitors; Roche). The samples were subsequently incubated on ice for 20 min followed by centrifugation at 16,250 × g at 4°C for 5 min. The supernatant was collected, and the amount of protein was then determined using Bradford reagent (Sigma-Aldrich Ltd.). The samples were stored at −80°C until further analysis.

### Western blot analysis for SOD, HO-1, CAT and GCS proteins

In order to measure the expression levels of SOD, HO-1, CAT and GCS, western blot analysis was used. In particular, whole cell lysate containing 50 *µ*g of protein was used for the determination of the SOD, HO-1 and CAT expression levels, while cytosolic lysate containing 30–50 *µ*g of protein was used for the determination of GCS levels. Cell lysates were separated by sodium dodecyl sulfate-polyacrylamide gel electrophoresis (SDS-PAGE) using an 8% polyacrylamide gel. Proteins were then transferred onto polyvinylidene difluoride (PVDF) membranes (Millipore, Bedford, MA, USA). The membranes were blocked overnight with 5% non-fat milk in 13 mM Tris/150 mM NaCl, pH 7.5, containing 0.2% Tween-20. They were then probed with polyclonal goat anti-human SOD-1 (1:1,600; Cat. no. sc-8634) or polyclonal rabbit anti-human GCS (1:1,600; Cat. no. sc-28965; both from Santa Cruz Biotechnology Inc., Dallas, TX, USA) or polyclonal goat anti-human HO-1 (1:1,400; Cat. no. AF3776) or polyclonal goat anti-human CAT (1:1,400; Cat. no. AF3398; both from R&D Systems, Minneapolis, MN, USA) primary antibodies for 1 h at room temperature. The membranes were then incubated with horseradish peroxidase-conjugated polyclonal goat anti-rabbit (1:5,000; Cat. no. 31462) or polyclonal donkey anti-goat (1:3,000; Cat. no. PA1-28659; both from Thermo Scientific, Rockford, IL, USA) secondary antibodies for 30 min at room temperature. All the membranes were re-probed with polyclonal rabbit anti-human (anti-mouse) glyceraldehyde 3-phosphate dehydrogenase (GAPDH; 1:1,000; Cat. no. PA1-988; Thermo Scientific) to normalize the data. The optical density of the protein bands was measured using Alpha View quantification software (Alpha Innotech, San Leandro, CA, USA). Each experiment was repeated 3 times.

### Determination of CAT activity

The determination of CAT activity in the whole cell lysate was carried out based on the method described in the study by Aebi (27). specifically, the reaction was carried out in a volume of 3 ml containing 150 *µ*l whole cell lysate and 2,845 *µ*l of 67 mM potassium phosphate buffer solution (pH 7.4). The measurement requires >30 *µ*g total amount of protein in the tested sample. The samples were incubated for 10 min at 37°C. Five microliters of 30% w/v H_2_O_2_ solution were added to the samples and the change in absorbance was immediately read at 240 nm (UV) for 1.5 min. CAT activity in the cell lysates was normalized to the total cellular protein level in each sample. The results are expressed as units (*µ*mol of H_2_O_2_ decomposed ml/min) per mg of protein. CAT activity was examined in at least 3 different lysates (each lysate was measured in triplicate).

### Determination of GST activity

The determination of GST activity in the cytosolic lysate was based on the method described in the study by Habig *et al* (28). More specifically, 920 *µ*l of phosphate buffer (100 mM, pH 7.4) were mixed with 50 *µ*l of GSH (1 mM) and 20 *µ*l of 1-chloro-2,4-dinitrobenzene (CDNB) and the samples were incubated for 5 min at 30°C. This was followed by the addition of 10 *µ*l of cytosolic lysate (the measurement requires >10 *µ*g of total amount of protein in the tested sample) and the change in absorbance was measured at 340 nm for 5 min. Upon conjugation of the thiol group of GSH to the CDNB substrate, there was an increase in the absorbance at 340 nm. The sample containing cytosolic lysate alone were used as the blank. GST activity in the cytosolic lysates was normalized to the total cellular protein level in each sample. The results are expressed as units (µmol of CDNB conjugate produced ml/min) per mg of protein. GST activity was examined in at least 3 different lysates (each lysate was measured in triplicate).

### Determination of SOD activity

The determination of SOD activity in the whole cell lysate was based on the method of nitroblue tetrazolium salt (NBT) as described in the study by Oberley and Spitz (29). More specifically, this assay included a negative control which was prepared by mixing 800 *µ*l of SOD buffer [1 mM diethylenetriaminepentaacetic acid (DETAPAC) in 0.05 M potassium phosphate buffer (pH 7.8), 1 unit CAT, 5.6×10^−5^ M NBT and 10^−4^ M xanthine] with 200 *µ*l of 0.05 M potassium phosphate buffer. Subsequently, ~60 mU of xanthine oxidase were added and the rate of increase in absorbance was measured at 560 nm for 3.5 min. In the test samples, 200 *µ*l of the total cell lysate (the measurement requires >10 *µ*g total amount of protein) were added to 800 *µ*l of SOD buffer followed by the addition of ~60 mU of xanthine oxidase and the rate of increase in absorbance was measured for 3.5 min at 560 nm. The calculation of SOD activity in the test samples is based on the percentage inhibition in the rate of increase in absorbance. The rate of increase in absorbance (*A*) per minute for the negative control and for the test samples was determined by the formula [1] and the percentage inhibition for each sample was calculated using the formula [2] as follows:

$$\Delta A_{560\mathrm{nm}}/\min=(A_{560\mathrm{nm}}\text{final-}A_{560\mathrm{nm}}\text{initial})/3.5\min$$ [1]

$$\%\text{Inhibitation}=[\left(\Delta A_{560\mathrm{nm}}/\min_{\text{negative}\;\text{control}}-\Delta A_{560\mathrm{nm}}/\min_{\text{sample}}\right)/\Delta A_{560\mathrm{nm}}/\min_{\text{negative}\;\text{control}}]\times 100$$ [2]

SOD activity in the whole cell lysates was normalized to the total cellular protein level in each sample. The results are expressed as units (one unit of SOD inhibits the rate of increase in absorbance at 550 nm by 50%) per mg of protein. SOD activity was examined in at least 3 different lysates (each lysate was measured in triplicate).

### Statistical analysis

All results are expressed as the means ± SD. For statistical analysis, one-way ANOVA was applied followed by Tukey’s test for multiple pair-wise comparisons. Dose-response relationships were examined by Spearman’s correlation analysis. Differences were considered statistically significant at P<0.05. All statistical analyses were performed using SPSS software (version 14.0; SPSS Inc., Chicago, IL, USA).

## Results

### Western blot analysis for SOD, HO-1, CAT and GCS protein expression

In order to examine the effects of GPE on the expression levels of antioxidant enzymes (i.e., SOD, HO-1, CAT and GCS), the C2C12 muscle cells were treated with GPE at concentrations of 2.5 and 10 *µ*g/ml. The EA.hy926 endothelial cells were treated with GPE at concentrations of 0.068 and 0.250 *µ*g/ml.

**Figure 1:**
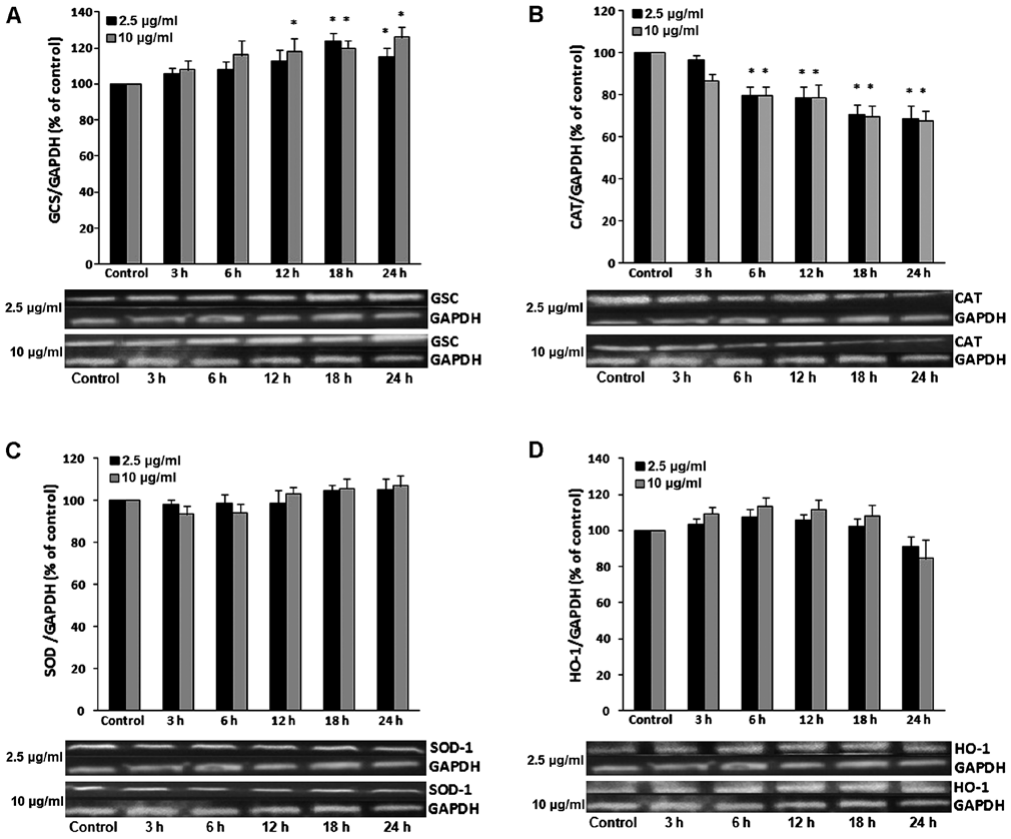
Representative western blots showing the effects of grape pomace extract (GPE) on the expression of (A) gamma-glutamylcysteine synthetase (GCS), (B) catalase (CAT), (C) superoxide dismutase (SOD) and (D) heme oxygenase 1 (HO-1) in C2C12 muscle cells. The results of densitometric quantification for all enzymes are also shown. The cells were incubated with GPE at 2.5 and 10 *µ*g/ml for 3, 6, 12, 18 and 24 h. The expression of GAPDH was used as a loading control for normalization. ^*^P<0.05, statistically significant difference compared to the control (untreated cells). The results are presented as the mean ± SEM.

**Figure 2:**
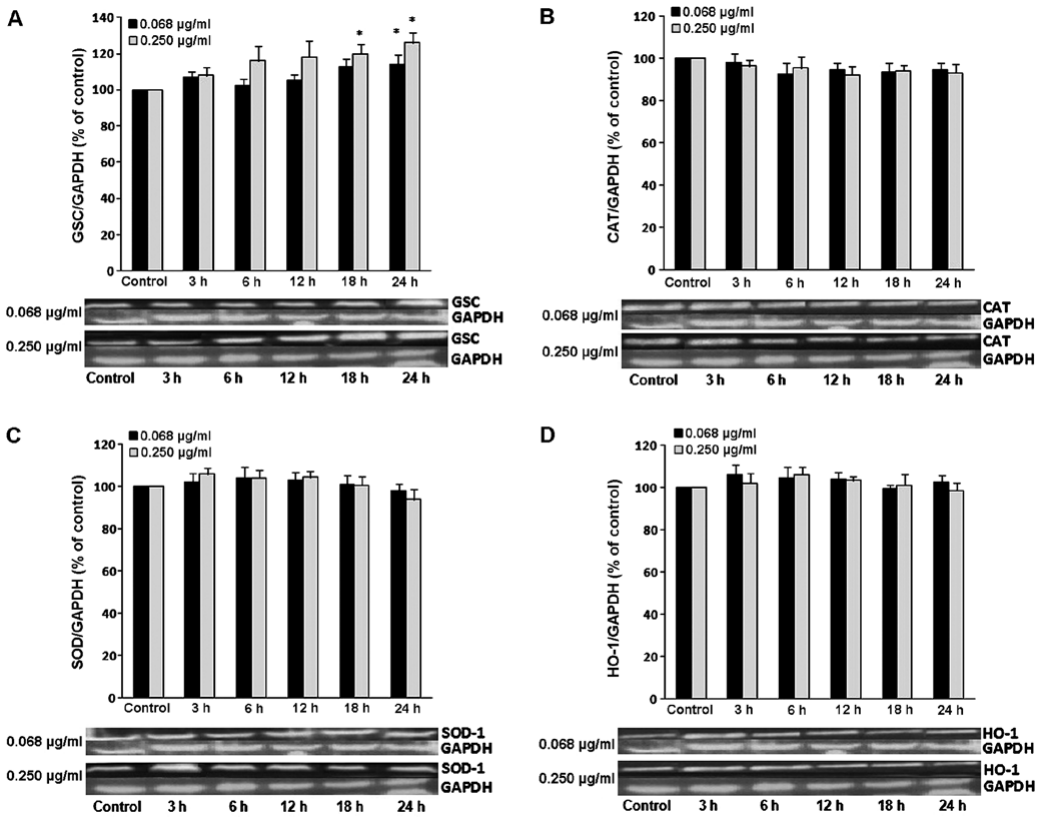
Representative western blots showing the effects of grape pomace extract (GPE) on the expression of (A) gamma-glutamylcysteine synthetase (GCS), (B) catalase (CAT), (C) superoxide dismutase (SOD) and (D) heme oxygenase 1 (HO-1) in EA.hy926 endothelial cells. The results of densitometric quantification for all enzymes are also shown. The cells were incubated with GPE at 0.068 and 0.250 *µ*g/ml for 3, 6, 12, 18 and 24 h. The expression of GAPDH was used as a loading control for normalization. ^*^P<0.05, statistically significant difference compared to the control (untreated cells). The results are presented as the means ± SEM.

Treatment with GPE at 2.5 *µ*g/ml significantly increased the GCS expression levels by 24.2 and 16.3% at 18 and 24 h, respectively compared to the control in the cytosolic lysate of C2C12 cells (Fig. 1A). However, treatment with GPE at 10 *µ*g/ml significantly increased the GCS levels in the C2C12 cells by 18.0, 20.3 and 26.1 at the 12, 18 and 24 h time points, respectively compared to the control (Fig. 1A). In the EA.hy926 endothelial cells, treatment with GPE at a concentration of 0.068 *µ*g/ml significantly increased the GCS levels by 14% at the 24 h time point, while treatment with GPE at the concentration of 0.250 *µ*g/ml led to a significant increase of 16.2% at 24 h time point compared to the control (Fig. 2A).

Moreover, the results revealed that treatment of the C2C12 cells with GPE at 2.5 *µ*g/ml significantly decreased the CAT expression levels by 20.4, 21.3, 29.2 and 31.5% at the 6, 12, 18 and 24 h time points, respectively, while treatment with GPE at 10 *µ*g/ml decreased the CAT levels by 20.7, 22.5, 30.3 and 32.3% at the 6, 12, 18 and 24 h time points, respectively compared to control in the total lysate of C2C12 cells (Fig. 1B). However, in the EA.hy926 cells, GPE did not significantly affect CAT expression at any concentration used (Fig. 2B).

Furthermore, the results revealed that none of the GPE concentrations used significantly affected SOD expression at any time point in the total lysate of C2C12 cells compared to the control (Fig. 1C). Similar results were observed in the EA.hy926 endothelial cells (Fig. 2C).

As observed with SOD expression, treatment with GPE did not significantly alter the HO-1 expression levels at any tested concentration at any time point compared to the control in the total lysates of both the C2C12 (Fig. 1D) and EA.hy926 cells (Fig. 2D).

### Assessment of GST activity

**Figure 3:**
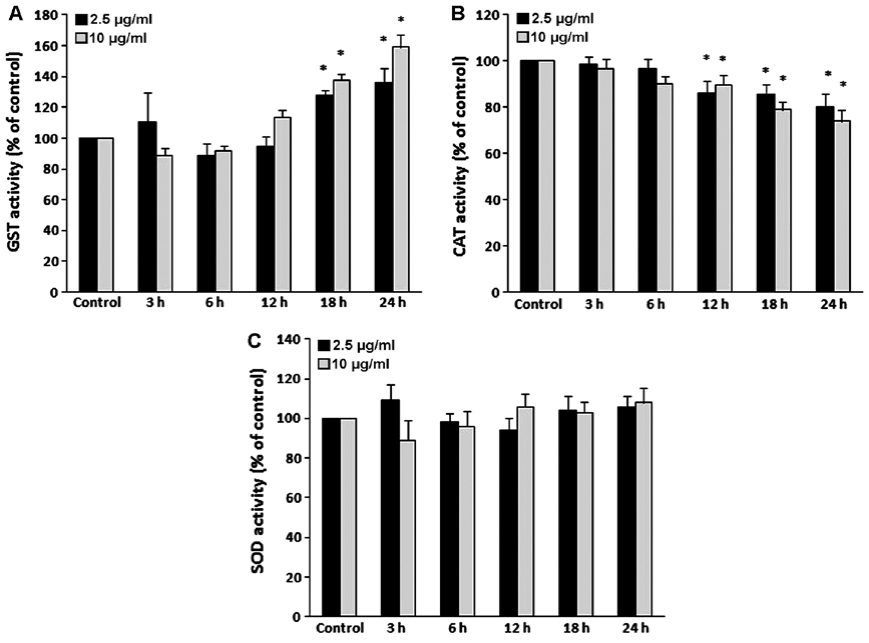
Effects of grape pomace extract (GPE) on the enzymatic activity of (A) glutathione S-tranferase (GST), (B) catalase (CAT) and (C) superoxide dismutase (SOD) in C2C12 muscle cells. The cells were incubated with GPE at 2.5 and 10 *µ*g/ml for 3, 6, 12, 18 and 24 h. The results are expressed as a percentage of the control values. ^*^P<0.05, statistically significant difference compared to the control (untreated cells). Results are presented as the means ± SEM.

In the C2C12 muscle cells, treatment with GPE at the concentration of 2.5 µg/ml significantly increased GST activity at the 18 and 24 h time points by 27.7 and 36.0%, respectively, while treatment with GPE at the concentration of 10 *µg/ml* increased GST activity by 37.7 and 59.0% at the 18 and 24 h time points, respectively compared to the control (Fig. 3A).

**Figure 4:**
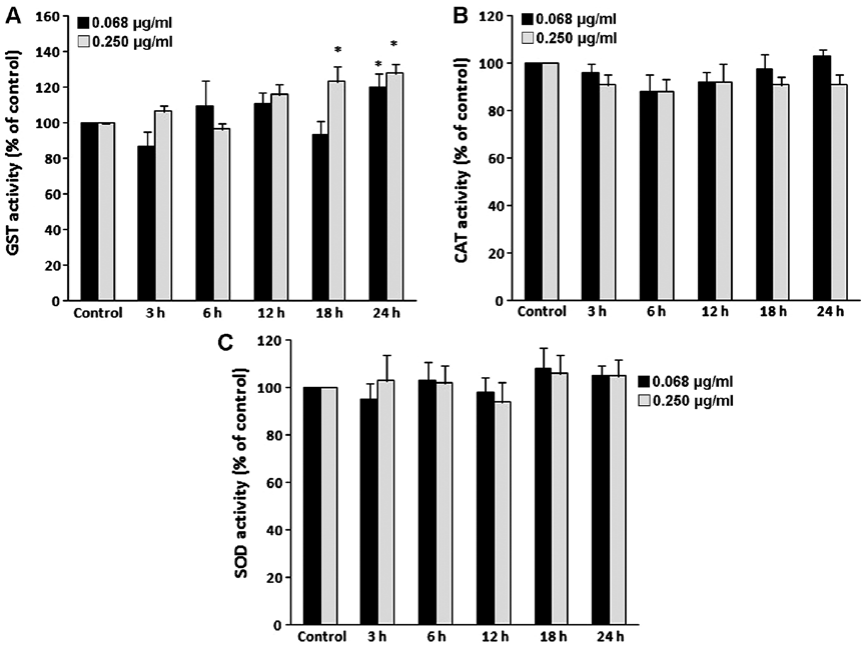
Effects of grape pomace extract (GPE) on the enzymatic activity of (A) glutathione S-tranferase (GST), (B) catalase (CAT) and (C) superoxide dismutase (SOD) in EA.hy926 endothelial cells. The cells were incubated with GPE at 0.068 and 0.250 µg/ml for 3, 6, 12, 18 and 24 h. The results are expressed as a percentage of the control values. ^*^P<0.05, statistically significant difference compared to the control (untreated cells). Results are presented as the means ± SEM.

In the EA.hy926 endothelial cells, treatment with GPE at 0.068 *µ*g/ml significantly increased GST activity at 24 h by 16.3%, while treatment with GPE at 0.250 *µ*g/ml increased GST activity by 23.3 and 28.1% at 18 and 24 h, respectively compared to the control (Fig. 4A).

### Assessment of CAT activity

In the C2C12 muscle cells, treatment with GPE at 2.5 *µg/ml* significantly decreased CAT activity by 12.7, 14.5 and 19.5% at the 12, 18 and 24 h time points, respectively compared to the control (Fig. 3B), while treatment with GPE at 10 *µ*g/ml decreased CAT activity by 8.3, 21.0 and 26.1% at the 12, 18 and 24 h time points, respectively compared to the control (Fig. 3B).

In the EA.hy926 endothelial cells, treatment with GPE at concentrations of 0.068 and 0.250 µ*g/ml* did not significantly affect CAT activity at any time point compared to the control (Fig. 4B).

### Assessment of SOD activity

In the C2C12 muscle cells, treatment with GPE did not significantly affect SOD activity at any concentration used at any time points compared to the control (Fig. 3C). Similar results were observed in endothelial cells (Fig. 4C).

## Discussion

In a previous study, we demonstrated that GPE reduced oxidative stress in endothelial and muscle cells (25). In the present study, in order to investigate the mechanisms through which these antioxidant effects are exerted, the effects of GPE on antioxidant enzymes and molecules were assessed in the EA.hy926 endothelial and C2C12 muscle cell lines. It should be noted that the GPE concentrations used were non-cytotoxic and very low, as in several studies on antioxidant compounds, high concentrations are used which are either difficult to be achieved in a human organism or they exhibit toxicity.

GSH, a tripeptide composed of glycine, cysteine and glutamic acid, is one of the most critical antioxidant molecules in cells and is involved in the detoxification of a number of xenobiotics and ROS through either the formation of S-conjugates or by serving as an electron donor from its sulfhydryl group (-SH) (30). Conjugation with GSH can occur both enzymatically and non-enzymatically. In human organisms, there are 3 main GSH systems: the GSH/glutathione peroxidase (GPx) system, which buffers H_2_O_2_ produced during cellular metabolism (30); the GSH/GST system, which conjugates GSH with xenobiotics for their detoxification (30); and the GSH/glutaredoxin (Grx) system which controls the cellular redox environment (31). In a previous study, we demonstrated that GPE increased GSH levels in EA.hy926 and C2C12 cells under either naive or oxidative stress conditions (25). This increase in GSH levels is explained by the GPE-induced increase in the expression of the GCS enzyme in both EA.hy926 and C2C12 cells. GCS is the first enzyme in the biosynthetic pathway of GSH, and consequently, it is critical for cell survival (32). It has also been reported that flavonoids increase intracellular GSH levels by the transactivation of the GCS catalytical subunit promoter (33). The importance of GSH for the antioxidant activity of GPE is also supported by the GPE-induced increase in GST activity. GST is induced under conditions of oxidative stress and is involved in the detoxification of organic epoxides, hydroperoxides and unsaturated aldehydes formed particularly after lipid peroxidation (34). GST detoxifies these products through their conjugation with GSH. Of note, we have previously reported that GPE decreased products of lipid peroxidation in EAhy.926 and C2C12 cells (25). Consequently, this effect may be attributed to the GPE-induced increase in GST activity. The increase in GST activity may result in a decrease in GSH levels (34). However, it seems that the GPE-induced increase in GCS levels led to *de novo* GSH synthesis that hampered the decrease in GSH levels caused by GST activity.

Based on the above-mentioned findings, it can be inferred that GSH systems play a crucial role in the antioxidant effects exerted by GPE in endothelial and muscle cells. Moreover, several studies conducted *in vivo* and using cell cultures have shown that grape seed extracts exert antioxidant effects through the induction of GSH systems in a great variety of tissues and organs, such as the liver, kidneys, heart, skin, pancreas, blood, eyes and brain (34–40). Although a large number of studies have indicated that grape extracts from seeds enhanced GSH systems, there are only few studies available that have used grape pomace extracts (34,37,41–46). Our results provide further evidence that grape extracts, in general, act as antioxidants through the modulation of GSH systems.

GPE did not seem to exert its antioxidant effects through the modulation of the other tested antioxidant enzymes (i.e., CAT, SOD and HO-1 enzymes). SOD converts O_2_^•−^ generated during oxidative stress into H_2_O_2_ (47). H_2_O_2_ may be converted to harmful ROS, but it is broken down into harmless water and oxygen by CAT (48). In this study, treatment with GPE reduced both the expression and activity of the CAT enzyme in endothelial cells, while in muscle cells, it had no any effect. GPE did not affect the expression or the activity of SOD in the EA.hy926 and C2C12 cells. The fact that GPE had no any effect (or even decreased) the expression and activity of CAT and SOD enzymes may be explained by its ability to enhance other antioxidant mechanisms, such as GSH, GCS and GST. Similar to our results, other studies have reported that grape extracts did not affect CAT and SOD enzymes (34,49). However, the administration of GPE to rats has been shown to increase CAT and SOD activity in the liver and kidneys (41). Furthermore, grape seed extracts have been shown to increase CAT and SOD activity in different tissues (35,40). These discrepancies between the results of different studies may be attributed to the different tissues used, as well as to the different chemical composition of the tested extracts. In addition Yang *et al* (49) reported that the effects of grape seed extract on CAT and SOD activity in eukaryotic cells depends on the presence or absence of oxidative stress stimulus and is mediated through the extracellular-signal-regulated kinase 1/2 (ERK1/2) signaling pathway.

HO-1 is also considered an important antioxidant enzyme (50). HO-1 is an ubiquitous inducible cellular stress protein and is the rate-limiting enzyme in the catabolism of heme to biliverdin, free iron and carbon monoxide. Biliverdin is rapidly converted to the strong antioxidant, bilirubin, which is then converted back into biliverdin through the reaction with ROS, leading to their neutralization (50). In this study, treatment with GPE did not affect HO-1 expression in the endothelial and muscle cells. Thus, the antioxidant effects of GPE do not seem to be mediated through HO-1 activity. Similar to our results, in a previous study, a grate seed extract rich in procyanidins was unable to increase HO-1 expression in liver cells (HepG2), although it induced the signaling pathway of nuclear factor E2-related factor (Nrf2)/antioxidant response element (ARE) (51). Nrf2/ARE is the main signaling pathway regulating HO-1 expression (52). In another study, the administration of grape extract to mice increased HO-1 expression levels through Nrf2 transcription factor in the testes (53). However, this grape extract was from seeds and was particularly rich in proanthocyanidins. Furthermore, resveratrol, one of the main polyphenols present in grape extracts, has been shown to increase HO-1 activity in mouse neuronal cells (54).

In conclusion, the findings of this study demonstrated that treatment with GPE exerted antioxidant effects in endothelial and muscle cells mainly through the induction of GCS and GST enzymes. These results, along with those of our our previous study (25), indicate that GPE increases GSH levels in EA.hy926 and C2C12 cells, suggesting the crucial role of GSH systems in the antioxidant effects of GPE. Thus, GPE may prove to be effective for use as a food supplement for the treatment of oxidative stress-induced pathological conditions of the cardiovascular and skeletal muscle systems, particularly those associated with low GSH levels. Although there are several studies showing that grape extracts from seeds protect cardiovascular and skeletal muscle systems from ROS-induced damage, there only few studies using extracts from pomace (40,55–63). Of course, *in vivo* studies are also required to confirm these findings.

## Abbreviations

GPE: grape pomace extract
GSH: glutathione
CAT: catalase
SOD: superoxide dismutase
HO-1: heme oxygenase 1
GCS: gamma-glutamylcysteine synthetase
GST: glutathione S-tranferase
ROS: reactive oxygen species
GAPDH: glyceraldehyde 3-phosphate dehydrogenase
H_2_O_2_: hydrogen peroxide
O_2_^•™^: superoxide radical anion
OH^•^: hydroxyl radical
RO^•^_2_: peroxyl radical
CDNB: 1-chloro-2,4-dinitrobenzene
NBT: nitroblue tretrazolium salt
DETAPAC: diethylenetriaminepentaacetic acid
DMEM: Dulbecco’s modified Eagle’s medium
FBS: fetal bovine serum
RIPA buffer: radio-immunoprecipication buffer
EDTA: ethylenediaminetetraacetic acid
HEPES: 4-(2-hydroxyethyl)-1-piperazineethane sulfonic acid

## References

[1] Valko M, Leibfritz D, Moncol J, Cronin MTD, Mazur M, Telser J (2007). Free radicals and antioxidants in normal physiological functions and human disease. Int J Biochem Cell Biol.

[2] Halliwell B, Wiley J, Sons (2001). Free radicals and other reactive species in disease. Nature Encyclopaedia of Life Sciences.

[3] Dupré-Croche S, Erard Mand Nüsse O (2013). ROS production in phagocytes: Why, when, and where?. J Leukoc Biol.

[4] Linnane AW, Zhang C, Yarovaya N, Kopsidas G, Kovalenko S, Papakostopoulos P, Eastwood H, Graves S, Richardson M (2002). Human aging and global function of coenzyme Q10. Ann NY Acad Sci.

[5] Mylonas C, Kouretas D (1999). Lipid peroxidation and tissue damage. In Vivo.

[6] Schneider BS, Tiidus PM (2007). Neutrophil infiltration in exercise-injured skeletal muscle: How do we resolve the controversy?. Sports Med.

[7] Nikolaidis MG, Kyparos A, Hadziioannou M, Panou N, Samaras L, Jamurtas AZ, Kouretas D (2007). Acute exercise markedly increases blood oxidative stress in boys and girls. Appl Physiol Nutr Metab.

[8] Meeus M, Nijs J, Hermans L, Goubert D, Calders P (2013). The role of mitochondrial dysfunctions due to oxidative and nitrosative stress in the chronic pain or chronic fatigue syndromes and fibromyalgia patients: Peripheral and central mechanisms as therapeutic targets?. Expert Opin Ther Targets.

[9] Phaneuf S, Leeuwenburgh C (2001). Apoptosis and exercise. Med Sci Sports Exerc.

[10] McClung JM, Deruisseau KC, Whidden MA, Van Remmen H, Richardson A, Song W, Vrabas IS, Powers SK (2010). Overexpression of antioxidant enzymes in diaphragm muscle does not alter contraction-induced fatigue or recovery. Exp Physiol.

[11] Deanfield JE, Halcox JP, Rabelink TJ (2007). Endothelial function and dysfunction: Testing and clinical relevance. Circulation.

[12] Hazel T, Müller T (2001). Culture of neuroepithelial stem cells. Curr Protoc Neurosci.

[13] Kokura S, Wolf RE, Yoshikawa T, Granger DN, Aw TY (1999). Molecular mechanisms of neutrophil-endothelial cell adhesion induced by redox imbalance. Circ Res.

[14] Rhee SG (2006). Cell signaling. H_2_O_2_, a necessary evil for cell signaling. Science.

[15] Woywodt A, Bahlmann FH, De Groot K, Haller H, Haubitz M (2002). Circulating endothelial cells: Life, death, detachment and repair of the endothelial cell layer. Nephrol Dial Transplant.

[16] Matthaiou CM, Goutzourelas N, Stagos D, Sarafoglou E, Jamurtas A, Koulocheri SD, Haroutounian SA, Tsatsakis AM, Kouretas D (2014). Pomegranate juice consumption increases GSH levels and reduces lipid and protein oxidation in human blood. Food Chem Toxicol.

[17] Samaras A, Tsarouhas K, Paschalidis E, Giamouzis G, Triposkiadis F, Tsitsimpikou C, Becker AT, Goutzourelas N, Kouretas D (2014). Effect of a special carbohydrate-protein bar and tomato juice supplementation on oxidative stress markers and vascular endothelial dynamics in ultra-marathon runners. Food Chem Toxicol.

[18] Kerasioti E, Kiskini A, Veskoukis A, Jamurtas A, Tsitsimpikou C, Tsatsakis AM, Koutedakis Y, Stagos D, Kouretas D, Karathanos V (2012). Effect of a special carbohydrate-protein cake on oxidative stress markers after exhaustive cycling in humans. Food Chem Toxicol.

[19] Bagchi D, Swaroop A, Preuss HG, Bagchi M (2014). Free radical scavenging, antioxidant and cancer chemoprevention by grape seed proanthocyanidin: An overview. Mutat Res.

[20] Apostolou A, Stagos D, Galitsiou E, Spyrou A, Haroutounian S, Portesis N, Trizoglou I, Wallace Hayes A, Tsatsakis AM, Kouretas D (2013). Assessment of polyphenolic content, antioxidant activity, protection against ROS-induced DNA damage and anticancer activity of Vitis vinifera stem extracts. Food Chem Toxicol.

[21] Spanou C, Veskoukis AS, Stagos D, Liadaki K, Anastasiadi M, Haroutounian SA, Tsouka M, Tzanakouli E, Kouretas D (2011). Effects of grape extracts on the in vitro activity of enzymes involved in oxidative stress regulation. In Vivo.

[22] Stagos D, Kazantzoglou G, Magiatis P, Mitaku S, Anagnostopoulos K, Kouretas D (2005). Effects of plant phenolics and grape extracts from Greek varieties of Vitis vinifera on mitomycin C and topoisomerase I-induced nicking of DNA. Int J Mol Med.

[23] Stagos D, Kazantzoglou G, Theofanidou D, Kakalopoulou G, Magiatis P, Mitaku S, Kouretas D (2006). Activity of grape extracts from Greek varieties of Vitis vinifera against mutagenicity induced by bleomycin and hydrogen peroxide in Salmonella typhimurium strain TA102. Mutat Res.

[24] Stagos D, Spanou C, Margariti M, Stathopoulos C, Mamuris Z, Kazantzoglou G, Magiatis P, Kouretas D (2007). Cytogenetic effects of grape extracts (Vitis vinifera) and polyphenols on mitomycin C-induced sister chromatid exchanges (SCEs) in human blood lymphocytes. J Agric Food Chem.

[25] Goutzourelas N, Stagos D, Demertzis N, Mavridou P, Karterolioti H, Georgadakis S, Kerasioti E, Aligiannis N, Skaltsounis L, Statiri A (2014). Effects of polyphenolic grape extract on the oxidative status of muscle and endothelial cells. Hum Exp Toxicol.

[26] Veskoukis AS, Kyparos A, Nikolaidis MG, Stagos D, Aligiannis N, Halabalaki M, Chronis K, Goutzourelas N, Skaltsounis L, Kouretas D (2012). The antioxidant effects of a polyphenol-rich grape pomace extract in vitro do not correspond in vivo using exercise as an oxidant stimulus. Oxid Med Cell Longev.

[27] Aebi H (1984). Catalase in vitro. Methods Enzymol.

[28] Habig WH, Pabst MJ, Jakoby WB (1974). Glutathione S-transferases. The first enzymatic step in mercapturic acid formation. J Biol Chem.

[29] Oberley LW, Spitz DR (1984). Assay of superoxide dismutase activity in tumor tissue. Methods Enzymol.

[30] Aquilano K, Baldelli S, Ciriolo MR (2014). Glutathione: New roles in redox signaling for an old antioxidant. Front Pharmacol.

[31] Lu J, Holmgren A (2014). The thioredoxin antioxidant system. Free Radic Biol Med.

[32] Dalton TP, Chen Y, Schneider SN, Nebert DW, Shertzer HG (2004). Genetically altered mice to evaluate glutathione homeostasis in health and disease. Free Radic Biol Med.

[33] Myhrstad MC, Carlsen H, Nordström O, Blomhoff R, Moskaug JØ (2002). Flavonoids increase the intracellular glutathione level by transactivation of the gamma-glutamylcysteine synthetase catalytical subunit promoter. Free Radic Biol Med.

[34] Fernández-Iglesias A, Quesada H, Díaz S, Pajuelo D, Bladé C, Arola L, Salvadó MJ, Mulero M (2014). Combination of grape seed proanthocyanidin extract and docosahexaenoic acid-rich oil increases the hepatic detoxification by GST mediated GSH conjugation in a lipidic postprandial state. Food Chem.

[35] Filip A, Daicoviciu D, Clichici S, Bolfa P, Catoi C, Baldea I, Bolojan L, Olteanu D, Muresan A, Postescu ID (2011). The effects of grape seeds polyphenols on SKH-1 mice skin irradiated with multiple doses of UV-B. J Photochem. Photobiol B.

[36] Janiques AG, Leal VO, Stockler-Pinto MB, Moreira NX, Mafra D (2014). Effects of grape powder supplementation on inflammatory and antioxidant markers in hemodialysis patients: A randomized double-blind study. J Bras Nefrol.

[37] Zhen J, Qu Z, Fang H, Fu L, Wu Y, Wang H, Zang H, Wang W (2014). Effects of grape seed proanthocyanidin extract on pentylenetetrazole-induced kindling and associated cognitive impairment in rats. Int J Mol Med.

[38] Yousef MI, Saad AA, El-Shennawy LK (2009). Protective effect of grape seed proanthocyanidin extract against oxidative stress induced by cisplatin in rats. Food Chem Toxicol.

[39] Zhang X, Hu Y (2012). Inhibitory effects of grape seed proanthocyanidin extract on selenite-induced cataract formation and possible mechanism. J Huazhong Univ Sci Technolog Med Sci.

[40] Saada HN, Said UZ, Meky NH, Abd El, Azime AS (2009). Grape seed extract Vitis vinifera protects against radiation-induced oxidative damage and metabolic disorders in rats. Phytother Res.

[41] Lakshmi BV, Sudhakar M, Aparna M (2013). Protective potential of black grapes against lead induced oxidative stress in rats. Environ Toxicol Pharmacol.

[42] Chen S, Zhu Y, Liu Z, Gao Z, Li B, Zhang D, Zhang Z, Jiang X, Liu Z, Meng L (2015). Grape seed proanthocyanidin extract ameliorates diabetic bladder dysfunction via the activation of the Nrf2 pathway. PLoS One.

[43] Chen Q, Zhang R, Li WM, Niu YJ, Guo HC, Liu XH, Hou YC, Zhao LJ (2013). The protective effect of grape seed procyanidin extract against cadmium-induced renal oxidative damage in mice. Environ Toxicol Pharmacol.

[44] Song Q, Shi Z, Bi W, Liu R, Zhang C, Wang K, Dang X (2015). Beneficial effect of grape seed proanthocyanidin extract in rabbits with steroid-induced osteonecrosis via protecting against oxidative stress and apoptosis. J Orthop Sci.

[45] Choi CS, Chung HK, Choi MK, Kang MH (2010). Effects of grape pomace on the antioxidant defense system in diet-induced hypercholesterolemic rabbits. Nutr Res Pract.

[46] Chidambara Murthy KN, Singh RP, Jayaprakasha GK (2002). Antioxidant activities of grape (Vitis vinifera) pomace extracts. J Agric Food Chem.

[47] Fridovich I (2011). Superoxide dismutases: Anti-versus pro-oxidants?. Anticancer Agents Med Chem.

[48] Kodydková J, Vávrová L, Kocík M, Žák A (2014). Human catalase, its polymorphisms, regulation and changes of its activity in different diseases. Folia Biol (Praha).

[49] Yang T, Li X, Zhu W, Chen C, Sun Z, Tan Z, Kang J (2014). Alteration of antioxidant enzymes and associated genes induced by grape seed extracts in the primary muscle cells of goats in vitro. PLoS One.

[50] Son Y, Lee JH, Chung HT, Pae HO (2013). Therapeutic roles of heme oxygenase-1 in metabolic diseases: Curcumin and resveratrol analogues as possible inducers of heme oxygenase-1. Oxid Med Cell Longev.

[51] Bak MJ, Jun M, Jeong WS (2012). Procyanidins from wild grape (Vitis amurensis) seeds regulate ARE-mediated enzyme expression via Nrf2 coupled with p38 and PI3K/Akt pathway in HepG2 cells. Int J Mol Sci.

[52] Jeong WS, Jun M, Kong AN (2006). Nrf2: A potential molecular target for cancer chemoprevention by natural compounds. Antioxid Redox Signal.

[53] Li SG, Ding YS, Niu Q, Xu SZ, Pang LJ, Ma RL, Jing MX, Feng GL, Liu JM, Guo SX (2015). Grape seed proanthocyanidin extract alleviates arsenic-induced oxidative reproductive toxicity in male mice. Biomed Environ Sci.

[54] Sakata Y, Zhuang H, Kwansa H, Koehler RC, Doré S (2010). Resveratrol protects against experimental stroke: Putative neuroprotective role of heme oxygenase 1. Exp Neurol.

[55] Luzak B, Kosiorek A, Syska K, Rozalski M, Bijak M, Podsedek A, Balcerczak E, Watala C, Golanski J (2014). Does grape seed extract potentiate the inhibition of platelet reactivity in the presence of endothelial cells?. Adv Med Sci.

[56] Luan SS, Yu F, Li BY, Qin RJ, Li XL, Cai Q, Yin WB, Cheng M, Gao HQ (2014). Quantitative proteomics study of protective effects of grape seed procyanidin B2 on diabetic cardiomyopathy in db/db mice. Biosci Biotechnol Biochem.

[57] Milenkovic D, Vanden Berghe W, Boby C, Leroux C, Declerck K, Szarc vel Szic K, Heyninck K, Laukens K, Bizet M, Defrance M (2014). Dietary flavanols modulate the transcription of genes associated with cardiovascular pathology without changes in their DNA methylation state. PLoS One.

[58] Badavi M, Abedi HA, Sarkaki AR, Dianat M (2013). Co-administration of grape seed extract and exercise training improves endothelial dysfunction of coronary vascular bed of STZ-induced diabetic rats. Iran Red Crescent Med J.

[59] Pajuelo D, Fernández-Iglesias A, Díaz S, Quesada H, Arola-Arnal A, Bladé C, Salvadó J, Arola L (2011). Improvement of mitochondrial function in muscle of genetically obese rats after chronic supplementation with proanthocyanidins. J Agric Food Chem.

[60] Ding Y, Dai X, Jiang Y, Zhang Z, Bao L, Li Y, Zhang F, Ma X, Cai X, Jing L (2013). Grape seed proanthocyanidin extracts alleviate oxidative stress and ER stress in skeletal muscle of low-dose streptozotocin- and high carbohydrate/high-fat diet-induced diabetic rats. Mol Nutr Food Res.

[61] Pajuelo D, Díaz S, Quesada H, Fernández-Iglesias A, Mulero M, Arola-Arnal A, Salvadó MJ, Bladé C, Arola L (2011). Acute administration of grape seed proanthocyanidin extract modulates energetic metabolism in skeletal muscle and BAT mitochondria nutrigenomics group. Agric Food Chem.

[62] Diaz MN, Frei B, Vita JA, Keaney JF (1997). Antioxidants and atherosclerotic heart disease. N Engl J Med.

[63] Rodriguez-Rodriguez R, Justo ML, Claro CM, Vila E, Parrado J, Herrera MD, Alvarez de Sotomayor M (2012). Endothelium-dependent vasodilator and antioxidant properties of a novel enzymatic extract of grape pomace from wine industrial waste. Food Chem.

